# Supplementary material for: Human-Based New Approach Methodologies in Developmental Toxicity Testing: A Step Ahead from the State of the Art with a Feto–Placental Organ-on-Chip Platform
Source: Int J Environ Res Public Health. 2022 Nov 28;19(23):15828. doi: 10.3390/ijerph192315828 (PMC9737555; doi:10.3390/ijerph192315828)
Supplement: Supplementary file 1 [file ijerph-19-15828-s001.zip › ijerph-2019635-supplementary.pdf]

Table S1. List of the AOPs present in the AOP-Wiki portal related to developmental toxicity and OECD TG 414, and grouped by affected system

System	Id	Title	AO	Life stage applicability	Taxonomy applicability	Sex applicability	Author	Status
**CNS**	12	Chronic binding of antagonist to N-methyl-D-aspartate receptors (NMDARs) during brain development leads to neurodegeneration with impairment in learning and memory in aging	Neurodegeneration; Learning and memory impairment	During brain development	Human, mouse, rat, monkey, zebrafish	NA	Florianne Tschudi-Monnet	Open for citation & comment
	13	Chronic binding of antagonist to N-methyl-D-aspartate receptors (NMDARs) during brain development induces impairment of learning and memory abilities	Learning and memory impairment	During brain development	Human, mouse, rat, monkey	Female, male	Anna Price	Open for citation & comment
	17	Binding of electrophilic chemicals to SH(thiol)-group of proteins and /or to seleno-proteins involved in protection against oxidative stress during brain development leads to impairment of learning and memory	Learning and memory impairment	During brain development	Human, mouse, rat	Female, male	Marie-Gabrielle Zurich	Under development: Not open for comment. Do not cite
	42	Inhibition of Thyroperoxidase and Subsequent Adverse Neurodevelopmental Outcomes in Mammals	Decreased cognitive function	During brain development	Human, mouse, rat	Female, male	Kevin Crofton	Open for citation & comment
	54	Inhibition of Na+/I- symporter (NIS) leads to learning and memory impairment	Learning and memory impairment	During brain development ; fetal; perinatal	Human, rat	Female, male	Anna Price	Open for citation & comment
	134	Sodium Iodide Symporter (NIS) Inhibition and Subsequent Adverse Neurodevelopmental Outcomes in Mammals	Decreased cognitive function	Perinatal	Human, rat	Female, male	Mary Gilbert	Under Development: Contributions and Comments Welcome
	152	Interference with thyroid serum binding protein transthyretin and subsequent adverse human neurodevelopmental toxicity	Decreased cognitive function	Development	Rat	Mixed	Kristie Sullivan	Under Development: Contributions and Comments Welcome
	300	Thyroid Receptor Antagonism and Subsequent Adverse Neurodevelopmental Outcomes in Mammals	Decreased cognitive function	During brain development	Human, mouse	Female, male	Kevin Crofton	Under development: Not open for comment. Do not cite

	405	Organo-Phosphate Chemicals induced inhibition of AChE leading to impaired cognitive function	Decreased cognitive function	All life stages	Human, mouse, rat	Female, male	Saroj Amar	Under development: Not open for comment. Do not cite
**Reproductive (*male*)**	18	PPARα activation in utero leading to impaired fertility in males	Impaired fertility; malformation	Development	Human, mouse, rat	Male	Elise Grignard	Open for citation & comment
	66	Modulation of Adult Leydig Cell Function Subsequent Glucocorticoid Activation in the Fetal Testis	Impaired fertility; decreased sperm quantity/quality	Fetal; Adult (reproductively mature)	Rat	Male	Gary Klinefelter	Under Development: Contributions and Comments Welcome
	67	Modulation of Adult Leydig Cell Function Subsequent to Estradiol Activation in the Fetal Testis	Impaired fertility; decreased sperm quantity/quality	Fetal; Adult (reproductively mature)	Rat	Male	Gary Klinefelter	Under Development: Contributions and Comments Welcome
	68	Modulation of Adult Leydig Cell Function Subsequent to Alterations in the Fetal Testis Protome	Impaired fertility; decreased sperm quantity/quality	Fetal; Adult (reproductively mature)	Rat	Male	Gary Klinefelter	Under Development: Contributions and Comments Welcome
	74	Modulation of Adult Leydig Cell Function Subsequent to Hypermethylation in the Fetal Testis	Impaired fertility; decreased sperm quantity/quality	Fetal; Adult (reproductively mature)	Rat	Male	Gary Klinefelter	Under Development: Contributions and Comments Welcome
	124	HMG-CoA reductase inhibition leading to decreased fertility	Impaired fertility	Fetal	Rat	Male	Kellie Fay	Under Development: Contributions and Comments Welcome
	288	Inhibition of 17α-hydrolase/C 10,20-lyase (Cyp17A1) activity leads to birth reproductive defects (cryptorchidism) in male (mammals)	Cryptorchidism	Development	Human, rat	Male	Bérénice Collet	Open for citation & comment
	305	5α-reductase inhibition leading to short anogenital distance (AGD) in male (mammalian) offspring	Decreased AGD	Pregnancy	Human, mouse, rat	Male	Terje Svingen	Under development: Not open for comment. Do not cite
	306	Androgen receptor (AR) antagonism leading to short anogenital distance (AGD) in male (mammalian) offspring	Decreased AGD	Pregnancy	Human, mouse, rat	Male	Terje Svingen	Under development: Not open for comment. Do not cite

	307	Decreased testosterone synthesis leading to short anogenital distance (AGD) in male (mammalian) offspring	Decreased AGD	Fetal; pregnancy	Human, mouse, rat	Male	Terje Svingen	Under development: Not open for comment. Do not cite
**Reproductive (*female*)**	167	Early-life estrogen receptor activity leading to endometrial carcinoma in the mouse	Endometrial carcinoma	Fetal to parturition; Juvenile	Mouse	Female	Charles Wood	Under Development: Contributions and Comments Welcome
	398	Inhibition of ALDH1A (RALDH) causing reduced all-trans retinoic acid levels leading to impaired fertility, females	Decreased fertility	During development and at adulthood	Human, mouse, rat	Female	Terje Svingen	Under development: Not open for comment. Do not cite
**Cardiovascular**	16	Acetylcholinesterase inhibition leading to acute mortality	Increased mortality	All life stages	NA	Unspecific	Dan Villeneuve	Under Development: Contributions and Comments Welcome
	43	Disruption of VEGFR Signaling Leading to Developmental Defects	Developmental defects	Conception to fetal; Pregnancy	Human, mouse, rat, zebrafish	NA	Tom Knudsen	Open for citation & comment
	104	Altered ion channel activity leading impaired heart function	Increased mortality	All life stages	NA	Unspecific	Kevin Dreher	Under Development: Contributions and Comments Welcome
	150	Aryl hydrocarbon receptor activation leading to early life stage mortality, via reduced VEGF	Early Life Stage Mortality	Embryo	Mouse, rat, zebrafish, chicken	Female, male	Amani Farhat	Open for citation & comment
	261	L-type calcium channel blockade leading to heart failure via decrease in cardiac contractility	Heart failure	All life stages	Vertebrates	Female, male	Luigi Margiotta-Casaluci	Under development: Not open for comment. Do not cite
	433	hERG inhibition leading to cardiac toxicity	Sudden cardiac death	All life stages	Human	Unspecific	Egemen Bilgin	Under development: Not open for comment. Do not cite
	436	Inhibition of RALDH2 causes reduced all-trans retinoic acid levels, leading to transposition of the great arteries	Transposition of the great arteries	Fetal	Mouse, vertebrates, chicken	Mixed	Gina Mennen	Open for comment. Do not cite

**Liver**	46	AFB1: Mutagenic Mode-of-Action leading to Hepatocellular Carcinoma (HCC)	Hepatocellular carcinoma	During development and at adulthood	Human, rat, monkey, dog, chickens	Unspecific	Ted Simon	Open for citation & comment
	107	Constitutive androstane receptor activation leading to hepatocellular adenomas and carcinomas in the mouse and the rat	Hepatocellular adenoma and carcinoma	All life stages	Mouse, rat	Female, male	Kristin Lichti-Kaiser	Open for citation & comment
	209	Perturbation of cholesterol and glutathione homeostasis leading to hepatotoxicity: Integrated multi-OMICS approach for building AOP	Hepatotoxicity	All life stages	NA	Unspecific	Jinhee Choi	Under development: Not open for comment. Do not cite
	220	Cyp2E1 Activation Leading to Liver Cancer	Liver cancer	All life stages	Human, rodents	Mixed	Francina Webster	Open for citation & comment
**Other soft organs**	39	Covalent Binding of Low Molecular Weight Organic Chemicals to Proteins leads to Sensitisation (Sensitization) of the Respiratory Tract	Allergic Respiratory Hypersensitivity Response	All life stages	Human, mouse	Unspecific	Kristie Sullivan	Under Development: Contributions and Comments Welcome
	206	Peroxisome proliferator-activated receptors γ inactivation leading to lung fibrosis	Lung fibrosis	All life stages	Human	Unspecific	Jinhee Choi	Under development: Not open for comment. Do not cite
	257	Receptor mediated endocytosis and lysosomal overload leading to kidney toxicity	Kidney toxicity	All life stages	Human, mouse, rat, monkey, dog	Unspecific	Angela Mally	Under development: Not open for comment. Do not cite
	298	Chronic reactive oxygen species leading to human treatment-resistant gastric cancer	Treatment-resistant gastric cancer	All life stages	Human	Unspecific	Shihori Tanabe	Open for comment. Do not cite
	316	Trypsin inhibition leading to pancreatic acinar cell tumors	Pancreatic acinar cell tumors	All life stages	Human, mouse, rat, macaca	Mixed	Shigeru Hisada	Under development: Not open for comment. Do not cite
**Skeletal**	94	Sodium channel inhibition leading to congenital malformations	Increased amputations	Fetal	Human, mouse, rat	NA	Kellie Fay	Under Development: Contributions and Comments Welcome
**Immune**	154	Inhibition of Calcineurin Activity Leading to Impaired T-Cell Dependent Antibody Response	Impairment of T-cell dependent antibody response	All life stages	Human, mouse, rat, macaca	Unspecific	Takumi Ohishi	Open for comment. Do not cite

	314	Binding to estrogen receptor (ER)-α in immune cells leading to exacerbation of systemic lupus erythematosus (SLE)	Exacerbation of systemic lupus erythematosus	All life stages	Human	Mixed	Yasuharu Otsubo	Under development: Not open for comment. Do not cite
	315	Inhibition of JAK3 leading to impairment of T-Cell Dependent Antibody Response	Impairment of T-cell dependent antibody response	All life stages	Human, mouse	Unspecific	Yasuhiro Yoshida	Under development: Not open for comment. Do not cite
**Others**	202	Inhibitor binding to topoisomerase II leading to infant leukaemia	Infant leukemia	Embryo	Human	Unspecific	Andrea Terron	Open for comment. Do not cite
	263	Uncoupling of oxidative phosphorylation leading to growth inhibition via decreased cell proliferation	Growth decrease	Embryo	Human, mouse, rat, zebrafish	Unspecific	You Song	Open for citation & comment
	265	Uncoupling of oxidative phosphorylation leading to growth inhibition via decreased lipid storage	Growth decrease	All life stages	NA	Unspecific	You Song	Under development: Not open for comment. Do not cite
	296	Oxidative DNA damage leading to chromosomal aberrations and mutations	Chromosomal aberrations increase	All life stages	Human, mouse, rat	Unspecific	Carole Yauk	Open for comment. Do not cite
**Placenta**	151	AhR activation leading to preeclampsia	Preeclampsia	Pregnancy	Human, mouse	Female	Sabrina Tait	Under development: Not open for comment. Do not cite
	431	Increased tumor necrosis factor (TNF) leading to increased risk of gestational diabetes mellitus (GDM)	Gestational diabetes mellitus	Pregnancy	Human	Female	Qi Wang	Under Development: Contributions and Comments Welcome
